# Therapeutic potential of inhalable medications to combat coronavirus disease-2019

**DOI:** 10.4155/tde-2020-0092

**Published:** 2020-11-17

**Authors:** Vineela Parvathaneni, Nishant S Kulkarni, Aaron Muth, Nitesh K Kunda, Vivek Gupta

**Affiliations:** ^1^College of Pharmacy & Health Sciences, St. John’s University, Queens, NY 11439, USA

**Keywords:** COVID-19, inhalation, non-invasive ventilation, pulmonary delivery, repurposing, vaccines

The recent outbreak of coronavirus disease-2019 (COVID-19) in Wuhan, China has rapidly spread across the world. The swift development of this pandemic has been attributed to ability of the Severe Acute Respiratory Syndrome-Coronavirus 2 (SARS-CoV-2) virus to spread from one person to another who are in close to proximity through droplet born transmission, typically by coughing or sneezing. According to the WHO dashboard, the total number of globally detected COVID-19 cases are >27 million with >891,000 deaths, as of 8 September 2020 [1]. SARS-CoV-2, the virus responsible for COVID-19 infections, is a mutated version of the novel severe acute respiratory syndrome coronavirus (SARS-CoV), which broke out in 2002. SARS-CoV-2 (a member of the Betacoronavirus genera of coronaviruses) causes respiratory tract infections, severe pneumonia, dry cough and even acute respiratory stress syndrome in some patients.

## Coronaviruses & typical implications of SARS-CoV-2

Coronaviruses are large (size: 125 nm, range between 60 and 140 nm), encased and ssRNA viruses. SARS-CoV-2 binds to the angiotensin converting enzyme-2 (ACE-2) receptor during host cell viral attachment. Through genetic recombination and variation, coronaviruses can rapidly adapt to and infect new hosts [2]. The parent SARS-CoV was associated with inflammation and severe lung damage via upregulation of pro-inflammatory cytokines such as IL-1/6/12, IFN-γ and MIP1A as well as others [3]. SARS-CoV-2 patients especially ones in the intensive care unit, have displayed high levels of pro-inflammatory cytokines, suggesting they play an important role in disease severity.

SARS-CoV-2 causes severe lower respiratory tract infection [3]. The majority of COVID-19 intensive care unit patients have presented severe complications due to respiratory failure, necessitating invasive mechanical ventilation to manage the disease. Additionally, more than 75% of patients hospitalized with COVID-19 have needed supplemental oxygen [2]. With the number of COVID-19 cases still rising worldwide, scientists are exploring all potential treatments; from vaccines to novel drug-delivery strategies to combat COVID-19. In addition, extensive investigations are underway into repurposing drugs which are already approved for a specific indication, for treating respiratory viral infections [4]. For instance, many US FDA-approved and investigational antiviral drugs used in the past against SARS and Middle East Respiratory Syndrome (MERS) (either alone or in combination), are now being investigated for their efficacy against COVID-19 [5].

It is crucial to properly comprehend virus–host immune responses and the pathological aspects of infection in finding effective therapies to prevent and/or regulate viral entry and replication, thereby diminishing its severe physiological effects. Several ongoing clinical trials include evaluation of the antiviral therapies, immune modulators and anticoagulants for efficacy against COVID-19. Despite these efforts, there is currently no treatment or vaccination available for COVID-19. Although, Sputnik-V vaccine was recently developed by Gamaleya National Center of Epidemiology and Microbiology (Moscow, Russia) demonstrating strong immune response, vaccine’s safety and efficacy could not be assured due to pending favorable Phase III results [6]. In order to maximize the efficacy of these investigational therapies for COVID-19, choosing a proper delivery system is vital to optimizing the dose accumulation and availability of the therapeutic at the site of infection, that is, deep lungs (predominant site of SARS-CoV-2 infection), without off-target distribution throughout the body. Body-wide distribution of a particular dose not only increases the dose required for therapeutic efficacy, but it also increases the chances of off-target side effects caused by the investigational medication. Currently, the best approach to manage disease conditions in the deeper areas of the respiratory tract involves aerosol delivery of medications that provide relief by localizing the dose deep in the lungs. Inhalation provides focused delivery of the drug as well as a vaccine to the lungs, resulting in a high degree of proper drug localization rather than a less specific systemic delivery. Moreover, when a considerable amount of a drug is localized to the target organ, the required dose is substantially decreased.

## Role of inhalation therapies in combating respiratory viral infections

In 2004, a pilot study reported that a low dose of inhaled nitric oxide could shorten the time of ventilator support for SARS-CoV infected patients [7]. Due to the genetic similarity of SARS-CoV-2 with its parent SARS-CoV, nitric oxide could be expected to successfully treat COVID-19 symptoms. Inhaled antiviral therapeutics have been established for treatment of influenza virus, echovirus, adenovirus, respiratory syncytial virus, coronavirus and others. For example, Ribavirin (Virazole^®^) is currently approved for the treatment of respiratory syncytial virus in neonates with underlying medical illness as well as preterm infants [8]. Currently, Virazole is in clinical trials to determine its efficacy against COVID-19 [8]. From a recent study, Sun *et al.* reported that inhaled remdesivir along with the current IV therapy may achieve enhanced efficacy against COVID-19 [9]. Similarly, hydroxychloroquine (HCQ), an FDA approved antimalarial drug, has demonstrated antiviral properties with potent anti-inflammatory activity (suppressing cytokines levels), thereby presenting a potential modality for COVID-19 treatment [10]. However, oral HCQ has exhibited a slow onset of action. To resolve this issue, Pulmoquine Therapeutics Inc. (Del Mar, CA, USA) is developing an aerosolized formulation of HCQ in order to achieve higher drug concentrations in epithelial airway cells than that observed with oral therapy, while also minimizing off-target effects [10]. Several other promising inhaled anti-infective drug candidates are currently under investigation, which could provide valuable therapeutic tools against several respiratory infectious diseases [11]. Iwabuchi *et al.* reported successful treatment of COVID-19 with ciclesonide (glucocorticoid) inhalation. The ability of inhaled steroids to alleviate localized inflammation in the lung as well as attenuate virus proliferation has been described in this study where three cases of mild-stage COVID-19 were treated [12]. Several other research groups have also been exploring the possibility of utilizing repurposed drugs/agents with antiviral activity as inhalations. For example, Synairgen has reported that an inhaled formulation of IFN-β is effective in lowering the risk of disease progression [13].

All previously mentioned inhalable medications need specific equipment and tools to make aerosol delivery possible. Aerosolized delivery of anti-infective agents can generally be carried out using a small-volume nebulizer, pressurized metered-dose inhaler (pMDI) or dry-powder inhaler [14]. While providing respiratory support and supplemental oxygen to COVID‐19 patients, one major short-coming of these devices is potential aerosolization of respiratory pathogens [15]. These patients exhale pathogen-containing aerosol droplets (bio-aerosols), that tend to linger in patient’s circumambient air and therefore, respiratory support for COVID‐19 patients must be selected by balancing the clinical benefits with the risks of nosocomial spread [15]. It has been reported that the escaped aerosol droplets can account for about 50% of total droplets and have the capability to survive in the air for a few hours. It has also been reported that SARS-CoV-2 remains viable and active in bio-aerosols and may be able to settle on nearby surfaces due to a patient sneezing or coughing [16]. Some aerosol-generating procedures may put healthcare workers at an increased risk for exposure to SARS-CoV-2 and infection via fugitive emissions during therapy. Therefore, these procedures are capable of producing fugitive emissions containing the virus to a larger extent than coughing, sneezing, etc. [16]. While there is no extensive literature available explaining the appropriate clinical practices in treating COVID-19 patients, Ari *et al.* summarized the potential practical strategies for aerosol delivery outside of protecting healthcare professionals from exposure to virus spread through exhaled droplets [14].

## Case studies elucidating strategies in managing risks associated with aerosol delivery

There are certain strategies that can be utilized to manage aerosol delivery in patients based on the severity and progression of their COVID-19 symptoms. Recently, A Ari [16], Ari* et al.* [14] and Fink *et al.* [17] have provided extensive literature on inhalation therapies while focusing on reducing the aerosol-related risk of COVID-19 transmission. In brief, A Ari, and Ari *et al.* summarized different strategies for aerosol-based therapeutic delivery to patients with COVID-19 based on the disease severity while also protecting healthcare providers from exposure to exhaled droplets during therapy [14,16]. Fink *et al.* summarized the specific recommendations for safe delivery of aerosol medications, while also highlighting the importance of using procedures with lower risks [17]. For patients with a mild progression of COVID-19, aerosol therapy should be avoided as it can cause viral transmission via bio-aerosols. If aerosol therapy is the only alternative, certain modifications can be performed, such as attaching high efficiency particulate air (HEPA) filters to nebulizers; or using jet or mesh nebulizers to help minimize bio-aerosol droplets. However, for patients requiring intensive care, the precautions and strategies to deliver medications safely differ from the sub-intensive or non-intensive cases. Patients with severe disease progression are usually suffering from respiratory failure, therefore nebulizer-based delivery is the only viable option for drug delivery. The use of mesh nebulizers has been recommended since they do not disrupt ventilator circuit flow, which is often the case with jet nebulizers. Along with these strategies, there are many factors that need to be considered while delivering aerosols to patients using mechanical ventilators. For example, pMDIs, which have been around for decades and serve as one of the most common ways to deliver aerosolized medication to patients on mechanical ventilators, require recognition of many factors which can affect the efficiency of aerosol delivery, such as time of actuation, priming and shaking the canister, actuator design, position of pMDIs in the ventilator circuit, heat and humidity of the room, and density of the inhaled gas. Similarly, multiple factors such as type of nebulizer, residual medication volume, correlation between bias flow and medication deposition and gas flow govern the delivery of aerosols utilizing a nebulizer [14].

It has been demonstrated that respiratory failures along with pneumonia and acute respiratory stress syndrome are hallmarks of severe COVID-19 pathology, and patients usually require artificial respiratory support or ventilation for survival while medications try to improve respiratory functioning. This ventilation can either be invasive, for example endotracheal intubation, or noninvasive, such as through a face mask or helmet assisted. Invasive procedures often lead to issues with patient compliance and distress. There has also been a lot of emphasis on developing novel techniques to improve noninvasive ventilation (NIV). One study by Patel *et. al.* involves the comparison of two types of NIV techniques, face mask (mask assisted NIV) versus helmet (helmet assisted NIV) [18]. It was reported that 61.5% of the patients utilizing a mask-assisted NIV underwent intubations whereas only 18.2% of patients on helmet-assisted NIV required intubation for respiratory support. There was a marked difference between ventilator free days for helmet using patients versus mask-assisted NIV, indicating that the helmet was better for managing NIV, thus concluding that the overall probability of patient survival was much higher for patients on helmet assisted NIV as compared with mask assisted NIV.

Another viable technique for providing patients with high levels of oxygen as well as constant respiratory support, is through high flow therapies such as high velocity nasal insufflation (HVNI), high flow oxygen, and high flow nasal cannula. These techniques have been criticized in the past for generation of high amounts of bio-aerosols. However, a study by Leonard *et. al.* assessed the feasibility in controlling bio-aerosols when using a mask assisted high flow therapy [19]. HVNI was used for therapy in three *in silico* simulated conditions, including HVNI with a mask, low flow oxygen delivery and no-therapy control. Simulation models revealed that HVNI with a mask managed to capture 83.2% of the particles being delivered and the rest of them were leaked through the intentional leaks from the masks. It has been hypothesized that the high flow of breathing was captured by the mask material and the slow diffusion of particles from the mask to the surrounding minimized the effect of airborne transmission of bio-aerosols. Similar results were obtained for simulations of low flow oxygen as well as no therapy, both with and without a mask, where mask-assisted simulation exhibited low degrees of bio-aerosol transmission.

Another study by Workman *et. al.* [20] reported an inexpensive way to control the risk of aerosolization stemming from endonasal intubation. A human cadaver was used to simulate intubation conditions and four tests were conducted using a fluorescently labeled solution to understand droplet behavior. These four studies utilized the cadaver with 4 sets of conditions: without a mask, with a normal surgical mask, with a perforated surgical mask which allows passage of an endoscope and with a specially designed low cost modified valved endoscopy of the nose and throat mask. The study was performed using three techniques mimicking endonasal intubation surgeries: nonpowered cold instrumentation, powered suction microdebrider and a high-speed drill. In all instances, it was reported that a normal surgical mask and the valved endoscopy of the nose and throat mask proved to be more effective in controlling contamination in the room with bio-aerosols as compared with no mask or a perforated surgical mask.

## A case for inhaled COVID-19 vaccine

Due to its extensive benefits and potential for effective delivery, the inhalation route has become a topic of interest for vaccine delivery as well. It has been predicted that COVID-19 may be a seasonal infection and as evidenced by the current number of cases and deaths worldwide, humanity cannot afford a COVID-19 reoccurrence. The only viable way to circumvent the reoccurrence is via acquired immunity for SARS-CoV-2. This acquired immunity could be provided by a vaccine that helps the body develop antibodies for SARS-CoV-2. Traditional vaccines, that is, parenteral vaccines are expensive, as they require trained medical personnel for administration, cold-chain dependence (refrigeration) to maintain stability and integrity of the antigen and complex manufacturing [21,22]. Most current vaccines require cold-chain storage and distribution to maintain its efficacy, in addition to requiring a visit to the clinic for immunization by trained medical personnel. Lack of infrastructure (cold-chain and trained medical personnel crucial for administering liquid-based vaccines) In low and middle-income countries can result in many children and adults not getting immunized [23]. These limitations severely restrict our ability to achieve mass vaccination in a global pandemic, such as the one we are currently facing. To meet the global demand of a vaccine, there is a dire need to develop noninvasive vaccine formulations that are effective, reliable, easily scalable, readily available as well as affordable. Issues with liquid-based vaccine formulations could be decreased through alternative formulations which can be administered via noninvasive routes of delivery or self-administered at home, and do not require cold-chain or trained medical personnel. Among all the noninvasive delivery routes, pulmonary administration, especially dry powder inhaler (DPI), deliver vaccines while overcoming the existing limitations of vaccination. Moreover, an inhaled vaccine will harness the potential of mucosal immune system in the lungs and high density of antigen-presenting cells, that is, alveolar macrophages (AMs), dendritic cells (DCs) and B cells in the lungs, to generate a strong mucosal and systemic immune response. Additionally, for SARS-CoV-2, the primary site of infection is the lungs – therefore an immune response generated in the lungs via a thermostable and inhalable vaccine administered into the lungs will be very advantageous.

The immune system in the respiratory tract consists of the epithelium at the surface; the immunocompetent cells in the underlying tissue (AMs, DCs, microfold [M] cells, intraepithelial lymphocytes); mucosa-associated lymphoid tissue and the lymph nodes draining the respiratory tract [24]. M cells facilitate transport of antigens from the mucus layer to the epithelial tissue which then passively diffuse into the underlying tissue. Then antigen-presenting cells (AMs and DCs), internalize the antigen, then process and present them to naive T cells (draining lymph nodes). The activated T cells then help B cells proliferate. At last, the CD4^+^ T cells help B cells to develop into plasma cells to produce neutralizing antibody titers.

## Conclusion

The current pandemic has resulted in the disruption of normalcy around the world, and there is an urgent need for developing and utilizing comprehensive approaches in treating COVID-19. While it is advantageous to use aerosol drug delivery, the risks associated with viral transmission due to distribution of viral pathogens in aerosol droplets have to be considered. Effective delivery of aerosolized medications to COVID-19 patients could be achieved with greater attention toward dose, frequency and the delivery technique selection during aerosol therapy. To ensure the safety of healthcare providers managing COVID-19, recent advancements in aerosol generation procedures and employment of surgical masks or helmets while providing respiratory support are crucial. However, there is need for aggressive innovations in developing newer aerosol-based technologies for COVID-19 treatment and prophylaxis (delivery of vaccines) to achieve safer and more effective management of the pandemic. Altogether, the evaluation of aerosol devices for possible contamination, and viral transmission; while providing strategies for their safe and effective use, are of paramount importance.

## Future perspective

Being a commentary article, it was not feasible to include all the recent work being conducted in the field of inhalational therapeutic delivery for COVID-19 treatment. For providing a more comprehensive review of the new development, we are currently working on articles discussing the nature of work currently underway and also a more comprehensive overview of device types, patient types, healthcare setting, etc. In addition, we will perform further work that includes a number of key areas around respiratory drug development, formulation and inhalation biopharmaceutics. Another interesting area that includes information about the treatment of respiratory infection via inhalation for other infectious agents, for example, aerosolized antibiotics like tobramycin or amikacin, will be presented in subsequent articles.
